# Treosulfan-Based Conditioning in Allogeneic Stem Cell Transplantation for Myelofibrosis: A Systematic Review

**DOI:** 10.3390/jcm15114005

**Published:** 2026-05-22

**Authors:** Abdulrahman Nasiri, Eman M. Nagiub, Mahmoud Aljurf, Mostafa F. Mohammed Saleh

**Affiliations:** 1 College of Medicine, Imam Mohammad Ibn Saud Islamic University (IMSIU), Riyadh 11432, Saudi Arabia; 2 Hematopathology Unit, Department of Clinical Pathology, Faculty of Medicine, Assiut University, Assiut 71515, Egypt; 3 Adult Hematology, Transplantation and Cellular Therapy Department, Oncology Center, King Faisal Specialist Hospital and Research Center, Riyadh 12713, Saudi Arabia; 4 Clinical Hematology Unit, Department of Internal Medicine, Faculty of Medicine, Assiut University, Assiut 71515, Egypt

**Keywords:** myelofibrosis, treosulfan, allogeneic stem cell transplantation, conditioning regimen, reduced-intensity conditioning, non-relapse mortality, busulfan, fludarabine

## Abstract

**Background**: Allogeneic hematopoietic stem cell transplantation (allo-HCT) is the only curative therapy for myelofibrosis (MF), but its use is limited by substantial transplant related morbidity and mortality, particularly in older or comorbid patients. Treosulfan has emerged as a less toxic alternative to busulfan, with potential advantages in myeloablative and reduced intensity conditioning. **Methods**: We conducted a comprehensive, multi-database literature search (PubMed, Scopus/EMBASE, Cochrane Library, Web of Science, and grey literature) for studies published between 2000 and 2025 evaluating treosulfan-based conditioning in MF patients undergoing allo-HCT. Data on patient characteristics, conditioning regimens, engraftment, graft-versus-host disease (GVHD), and survival outcomes were synthesized. **Results**: Eight studies including more than 800 patients were analyzed. Treosulfan was most commonly combined with fludarabine, with or without additional agents. Engraftment rates were consistently high at 94 to 100%, with low non-relapse mortality (NRM) and favorable progression-free survival (PFS). An EBMT registry study demonstrated superior survival and significantly lower NRM compared with busulfan based regimens. Benefits were observed across older patients, alternative donors, and second transplants. Higher treosulfan doses were associated with increased toxicity in some cohorts. **Conclusions**: Treosulfan based conditioning offers an effective and better tolerated option for MF transplantation. Prospective trials are needed to refine dosing and patient selection.

## 1. Introduction

Myelofibrosis (MF) is a chronic BCR-ABL1-negative myeloproliferative neoplasm characterized by bone marrow fibrosis, cytopenias, extramedullary hematopoiesis, and a risk of leukemic transformation. Allogeneic hematopoietic stem cell transplantation (allo-HCT) remains the only curative option for patients with MF, particularly those with intermediate-2 or high-risk disease per DIPSS or MYSEC-PM criteria. However, transplant-related morbidity and mortality remain considerable, particularly in older patients and those with comorbidities. The development of reduced-intensity conditioning (RIC) regimens has aimed to expand transplant eligibility, but these regimens must balance lower toxicity with sufficient disease control and durable engraftment [1].

Treosulfan is a bifunctional alkylating agent with both immunosuppressive and myeloablative properties. It has garnered increasing interest as a conditioning agent due to its favorable toxicity profile, predictable pharmacokinetics, and reduced organ toxicity compared to busulfan. Treosulfan functions as a prodrug that undergoes non-enzymatic conversion to active monoepoxides and diepoxides, resulting in DNA cross-linking and cell death. Unlike busulfan, treosulfan demonstrates lower hepatotoxicity and reduced risk of veno-occlusive disease (VOD), making it particularly attractive for patients with pre-existing organ dysfunction or advanced age [2,3,4].

When combined with fludarabine, treosulfan-based conditioning has shown promising results across various hematologic malignancies, including MF. Studies have demonstrated that treosulfan regimens offer reliable engraftment, low non-relapse mortality (NRM), and overall survival (OS) rates that are comparable or superior to busulfan-based approaches, especially in older or medically vulnerable populations. The flexibility of treosulfan dosing allows for adaptation to both myeloablative and reduced-intensity platforms, providing clinicians with a versatile tool for individualized transplant strategies [5].

Given the growing body of evidence supporting treosulfan use and the increasing number of studies evaluating its role in MF, a systematic synthesis of the available literature is warranted. This review aims to comprehensively summarize current evidence on the safety and efficacy of treosulfan-based conditioning regimens in allogeneic transplantation for myelofibrosis, comparing outcomes across various patient populations, donor types, and treatment strategies to inform future clinical practice and guide research priorities.

## 2. Methods

**Figure 1:**
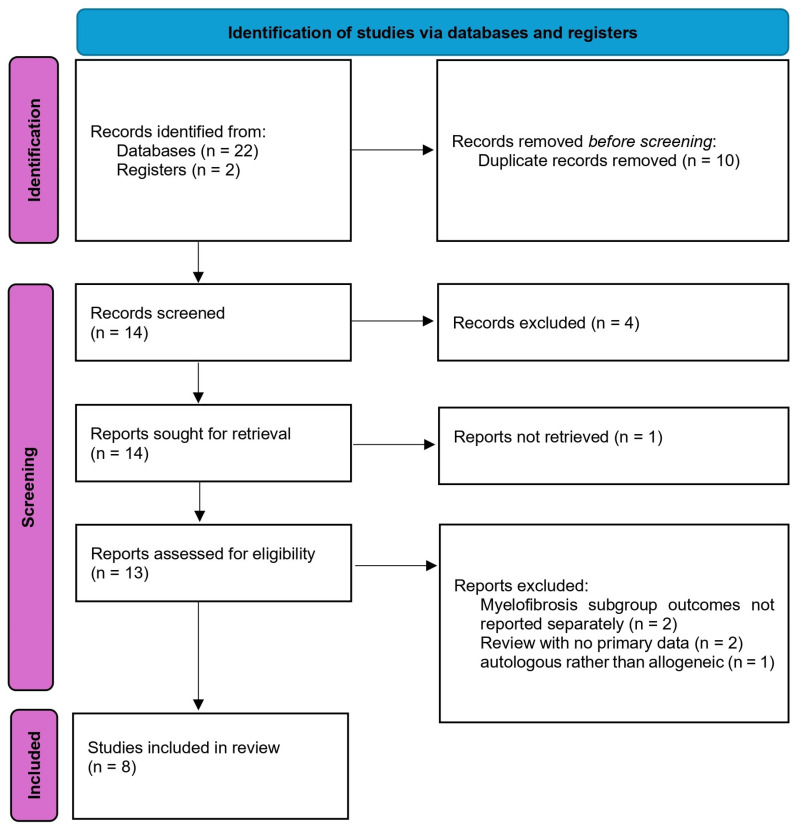
PRISMA 2020 Flow Diagram.

We conducted a comprehensive, multi-database literature search (PubMed, Scopus/EMBASE, Cochrane Library, Web of Science, and grey literature) for studies published between 2000 and 2025 evaluating treosulfan-based conditioning in MF patients undergoing allo-HCT. The search strategy and study selection process were documented in accordance with PRISMA 2020 guidelines, using a completed PRISMA 2020 flow diagram (Figure 1), and the completed PRISMA 2020 checklist is provided as Supplementary Table S1. The review protocol was not prospectively registered in a public registry. The search focused on articles published between January 2000 and December 2025 and utilized combinations of the following keywords: “Treosulfan,” “myelofibrosis,” “conditioning regimen,” “allogeneic stem cell transplantation,” and “hematopoietic cell transplantation,” with Boolean operators (AND/OR) employed to refine results. To ensure comprehensive coverage, additional relevant studies were identified by examining the reference lists of included articles. The search focused on English-language articles.

Eligible studies included those that reported on adult or pediatric patients with myelofibrosis who underwent allo-HCT using treosulfan-based conditioning regimens, either as monotherapy or in combination with other agents at any dose. Inclusion required reporting of at least one key transplant-related outcome such as overall survival (OS), progression-free survival (PFS), relapse rate, or non-relapse mortality (NRM) specific to the treosulfan cohort. Both prospective and retrospective studies, including registry-based analyses, were considered. Studies were excluded if they focused on other underlying diseases or did not provide outcome data specific to treosulfan use. In cases where pediatric studies included small treosulfan cohorts without isolated outcomes, they were retained solely to illustrate the feasibility of the regimen in rare subpopulations, though their analytical weight is limited.

From each selected study, data were extracted regarding study design, patient characteristics, conditioning protocols, donor type, engraftment, graft-versus-host disease (GVHD) incidence, and transplant outcomes (OS, PFS, relapse, NRM). The quality and relevance of studies were assessed qualitatively. Due to variability in study design and outcome reporting, findings are presented in a descriptive, narrative format rather than a pooled meta-analytic synthesis. We acknowledge the potential for overlapping patient populations across large registry studies (e.g., EBMT cohorts); therefore, patient numbers are discussed per study rather than cumulatively.

## 3. Results

A total of eight studies met the inclusion criteria, encompassing data on more than 800 patients with myelofibrosis who received treosulfan-based conditioning regimens prior to allo-HCT. The studies varied in design and population, including registry-based analyses, single-center retrospective cohorts, and prospective case series, and included adult, elderly, and pediatric patients (Table 1). Treosulfan was most commonly used in combination with fludarabine, often with additional agents such as thiotepa, melphalan, or cyclophosphamide, and was employed in both matched and alternative donor settings. To facilitate comparison, results are grouped thematically by study scope and patient population.

Across studies, engraftment rates were consistently high, relapse incidence was generally low, and non-relapse mortality (NRM) was acceptable, particularly in reduced-intensity regimens. While outcomes varied depending on disease status, donor type, and treosulfan dose intensity, the collective data support the feasibility and safety of treosulfan conditioning in myelofibrosis patients, especially in those requiring reduced-toxicity regimens or with high-risk clinical features.

### 3.1. Large Registry and Multicenter Studies

Robin et al. conducted a large EBMT registry study comparing outcomes in 530 MF patients who underwent allo-HCT with either treosulfan-based (*n* = 73) or busulfan-based (*n* = 457) conditioning regimens [4]. The busulfan cohort was stratified into high-dose (BU-HD, >6.4 mg/kg) and reduced-intensity (BU-RIC, ≤6.4 mg/kg) groups. Treosulfan demonstrated significantly superior progression-free survival compared to both BU-HD (HR 0.57, *p* = 0.003) and BU-RIC (HR 0.60, *p* = 0.006). Overall survival was also significantly better with treosulfan compared to BU-HD (HR 0.61, *p* = 0.01), though the difference with BU-RIC did not reach statistical significance. Notably, NRM was significantly lower in the treosulfan group compared to BU-HD (HR 0.44, *p* = 0.003), while relapse rates were similar across all groups. While these findings suggest a favorable safety profile for treosulfan, the retrospective nature of the study necessitates cautious interpretation of comparative superiority [4].

Gagelmann et al. reported outcomes from a cohort of 1115 MF patients, of whom 213 received treosulfan-based conditioning [3]. The study compared reduced-intensity treosulfan (RIC-TREO) with both reduced-intensity and high-dose busulfan regimens. The 4-year OS in the RIC-TREO group was 69%, with relapse risk comparable to busulfan-based regimens. However, a critical finding was that higher treosulfan doses (≥42 g/m^2^) were associated with significantly increased NRM (HR 1.61, *p* = 0.006), emphasizing the importance of dose optimization. In contrast, reduced-intensity treosulfan doses (<42 g/m^2^) were associated with favorable outcomes and acceptable toxicity, supporting the use of lower-dose treosulfan in the RIC setting [3].

Polverelli et al. conducted an Italian multicenter retrospective analysis of 37 MF patients receiving allo-HCT with treosulfan-based double-alkylator regimens, predominantly treosulfan combined with thiotepa or melphalan [6]. The cohort included high-risk patients, with 70.2% having an Eastern Cooperative Oncology Group (ECOG) performance status ≥1 and 37.8% with high hematopoietic cell transplantation-comorbidity index (HCT-CI) scores. Despite these adverse features, engraftment was achieved in 94.6% of patients. The 1-year OS was 62.2%, and PFS was 59.6%. The 1-year cumulative incidence of NRM was 33.5%, while relapse incidence was 14.5%. Acute GVHD grade II-IV occurred in 21.6% of patients, and chronic GVHD in 18.8%. The regimen was generally well tolerated, though higher treosulfan doses (≥47 g/m^2^) were associated with increased early toxicity and NRM, consistent with findings from other studies [6].

### 3.2. Second Transplantation and Salvage Settings

Atagunduz et al. evaluated outcomes in 33 patients with relapsed MF who underwent second allogeneic HCT using treosulfan-based conditioning (36–42 g/m^2^) combined with fludarabine, with or without antithymocyte globulin (ATG) [8]. This represents a particularly challenging clinical scenario, as second transplants are associated with higher risks of toxicity and treatment failure. Despite these challenges, the study reported a 3-year OS of 59% and a 5-year disease-free survival (DFS) of 45%. The 5-year cumulative incidence of relapse was 16%, and day-100 NRM was 16%, increasing to 31% at 3 years. Moderate-to-severe chronic GVHD occurred in 28% of patients. These results demonstrate that treosulfan-based conditioning is feasible and effective in the salvage setting, offering a reasonable chance of long-term disease control even after prior transplant failure [8].

### 3.3. Single-Center and Pilot Studies

Claudiani et al. described outcomes in 14 MF patients who received treosulfan-based reduced-toxicity conditioning followed by allo-HCT [10]. The cohort included 10 patients with matched related donors (MRD) and 4 with matched unrelated donors (MUD). The conditioning regimen consisted of treosulfan plus fludarabine, with some patients receiving additional low-dose total body irradiation (TBI) or ATG. The study reported 100% engraftment, a 3-year OS of 54%, and a 3-year PFS of 46%. However, 2-year NRM was 39%, particularly higher in MUD recipients, reflecting the increased immunologic challenges associated with unrelated donor transplantation. Acute GVHD grade II-IV occurred in 50% of patients, and chronic GVHD in 48%, rates that are consistent with the use of unrelated donors and reduced-intensity conditioning [10].

Campodonico et al. conducted a pilot study at San Raffaele Hospital that included 14 MF patients (median age 56.5 years) who underwent allo-HCT after pre-transplant splenic irradiation followed by treosulfan/fludarabine conditioning, with or without melphalan [11]. Splenomegaly is a common complication in MF and can impair engraftment and increase transfusion requirements. In this study, post-transplant spleen size decreased by a median of 25%, demonstrating the efficacy of combined splenic irradiation and treosulfan conditioning. Neutrophil engraftment occurred in 13 of 14 patients, with primary graft failure (PGF) occurring in 5 patients, 4 of whom subsequently recovered. At 36 months, 9 patients were alive and in remission. Acute GVHD grade II-IV occurred in 8 patients, and chronic GVHD in 7. Treosulfan was well tolerated with acceptable NRM, supporting its use in combination with splenic irradiation for patients with significant splenomegaly [11].

### 3.4. Elderly Patients

Gagelmann et al. conducted a German multicenter registry analysis of 115 MF patients aged ≥ 70 years who underwent allo-HCT. Of these, 19 patients (17%) received treosulfan plus fludarabine conditioning [7]. Outcomes in the treosulfan group were comparable to those achieved with busulfan-based regimens, with 96% engraftment and a 1-year relapse incidence of only 7%. These findings support the use of treosulfan in older patients, a population in whom reduced toxicity is of paramount importance. The favorable engraftment and low relapse rates suggest that treosulfan provides adequate disease control without excessive toxicity in this vulnerable demographic [7].

### 3.5. Pediatric Populations

Wachowiak et al. reported outcomes from an EBMT Paediatric Diseases Working Party retrospective study of 35 pediatric patients with myelofibrosis [9]. Treosulfan-based conditioning was used in 6 patients (17.1%), predominantly in non-MSD transplants. Regimens included combinations of treosulfan with fludarabine, thiotepa, cyclophosphamide, or melphalan. Although specific outcomes for the treosulfan subgroup were not reported separately, the use of treosulfan in this cohort suggests that it is considered a feasible and well-tolerated option in pediatric MF, a rare but challenging clinical entity where long-term toxicity is a critical concern [9].

## 4. Discussion

Treosulfan has emerged as a compelling alternative to traditional alkylating agents such as busulfan in conditioning regimens for patients with myelofibrosis undergoing allogeneic hematopoietic stem cell transplantation. The accumulating evidence supports its favorable toxicity profile, consistent engraftment outcomes, and sustained disease control across diverse transplant settings. The large EBMT registry analysis by Robin et al. provides the most robust comparative data to date, demonstrating a significant advantage in progression-free survival and overall survival with treosulfan-based conditioning compared to both high-dose and reduced-intensity busulfan regimens, along with substantially reduced non-relapse mortality (HR 0.44 vs. BU-HD) and comparable relapse rates [4]. Collectively, these findings support treosulfan-based conditioning as a clinically relevant and increasingly attractive first-line option for patients with myelofibrosis undergoing allo-HCT, while recognizing that definitive conclusions regarding superiority require prospective validation.

The survival benefit observed with treosulfan may be partly attributable to its reduced extramedullary toxicity. Unlike busulfan, which is associated with hepatic veno-occlusive disease, pulmonary complications, and seizures, treosulfan exhibits lower organ toxicity due to its prodrug nature and more predictable pharmacokinetics [3,10]. Supporting this mechanistic advantage, Remberger et al. demonstrated in a prospective toxicity analysis of 118 allo-HCT recipients that treosulfan/fludarabine conditioning was associated with only mild and transient hepatic enzyme elevations, rare veno-occlusive disease, and low early non-relapse mortality (7.5% at day 100 and 11.9% at 1 year), despite myeloablative-level marrow toxicity. These findings provide biological plausibility for the reduced regimen-related mortality observed in large MF transplant cohorts and reinforce treosulfan’s role as a reduced-toxicity conditioning platform [12].

The favorable outcomes seen with treosulfan are not limited to MF alone. A large meta-analysis by Zhu et al. (2020), which included nearly 4000 patients with myelodysplastic syndromes (MDS) or acute myeloid leukemia (AML), showed that treosulfan-based conditioning was associated with significantly improved overall survival (HR = 0.80) compared to busulfan, while maintaining similar relapse rates and non-relapse mortality [2]. Notably, treosulfan was also associated with a significantly lower risk of acute GVHD (HR = 0.70), further strengthening its appeal as a reduced-toxicity alternative in myeloid malignancies.

The role of treosulfan in challenging clinical contexts such as second allo-HCT and patients with large spleens has been further explored in smaller cohort studies. Atagunduz et al. demonstrated the feasibility of treosulfan-based conditioning in relapsed MF patients undergoing a second transplant, achieving a 3-year OS of 59% and low relapse rates, despite the anticipated high-risk features of this cohort [8]. Similarly, a single-center study incorporating splenic irradiation (SI) before treosulfan conditioning reported meaningful reductions in spleen size and acceptable engraftment, even among patients with prior primary graft failure (PGF) [11]. These findings highlight treosulfan’s potential as part of a multimodal approach to MF transplantation, particularly in patients where spleen volume is a barrier to successful engraftment.

The toxicity-sparing potential of treosulfan also makes it a logical option for elderly patients or those with significant comorbidities. In a German multicenter registry study involving patients aged ≥ 70 years, Gagelmann et al. reported excellent engraftment rates (96%) and low 1-year relapse incidence (7%) in the treosulfan/fludarabine group [7]. These outcomes, comparable to those achieved with busulfan, support the inclusion of treosulfan in reduced-toxicity regimens for geriatric or frail patients, a growing demographic in hematologic transplantation.

In pediatric MF a rare but challenging entity treosulfan’s role is still evolving. The EBMT pediatric cohort by Wachowiak et al. included a small subset of patients who received treosulfan-based regimens, particularly in unrelated donor or haploidentical transplant settings. Although detailed subgroup outcomes were not reported, the use of treosulfan in this setting reflects clinical confidence in its safety for children, where long-term toxicity is a paramount concern [9]. Supporting this, a recent systematic review and meta-analysis by Wu et al. analyzed six studies comparing treosulfan-versus busulfan-based conditioning in pediatric HSCT and found no significant differences in the incidence of acute GVHD (OR 0.96), grade II–IV acute GVHD (OR 1.19), chronic GVHD (OR 1.18), veno-occlusive disease (OR 0.92), or transplant-related mortality (OR 0.70). Interestingly, there was a marginal survival benefit associated with treosulfan (OR 1.57; 95% CI 1.00–2.44), although this was not stable on sensitivity analysis [13]. These findings reinforce the tolerability of treosulfan in pediatric populations and highlight the need for prospective randomized studies to validate potential survival advantages and confirm long-term safety profiles.

One emerging area of interest is the combination of treosulfan with agents such as thiotepa or melphalan. While thiotepa has demonstrated synergistic cytoreduction and CNS penetration in other hematologic malignancies, its impact in MF remains understudied. Some regimens have incorporated thiotepa or cyclophosphamide in addition to treosulfan/fludarabine, with acceptable tolerability, though definitive evidence comparing these combinations is lacking [9,10]. The recent Italian multicenter retrospective study by Polverelli et al. (2025) provides valuable insight into this area, evaluating 37 patients with MF who received treosulfan-based double-alkylator regimens (primarily treosulfan with thiotepa or melphalan) [6]. Despite inclusion of high-risk patients including 70% with ECOG ≥ 1 and over one-third with high HCT-CI engraftment was successful in 94.6%, and relapse incidence remained low (14.5%). However, the 1-year NRM was notably elevated at 33.5%, particularly among those receiving higher treosulfan doses (≥47 g/m^2^), reinforcing concerns raised in earlier reports regarding dose-dependent toxicity. Nevertheless, rates of acute and chronic GVHD were relatively low, suggesting potential synergy between treosulfan’s immunomodulatory profile and GVHD prophylaxis strategies.

Additionally, donor type remains an important variable. While matched sibling donor (MSD) settings consistently yield lower NRM, studies such as Claudiani et al. report elevated 2-year NRM (39%) among matched unrelated donor (MUD) recipients conditioned with treosulfan [10]. These findings suggest that treosulfan-based regimens may not fully mitigate the risks associated with HLA mismatches or unrelated grafts, highlighting the importance of GVHD prophylaxis and supportive care optimization in these cases.

Post-transplant strategies, such as maintenance therapies and measurable residual disease (MRD)-guided approaches, are critical considerations that warrant further exploration. While current data on treosulfan conditioning primarily focus on early transplant outcomes, the integration of post-HCT targeted therapies (e.g., JAK inhibitors or hypomethylating agents) may further reduce relapse risk and improve long-term survival, representing an important avenue for future research.

Several limitations of this systematic review should be acknowledged. The evidence base relies predominantly on retrospective and registry-derived studies, which introduces unavoidable heterogeneity, confounding, and selection bias. The small sample sizes reported in pilot and pediatric cohorts, together with the possibility of overlapping patients across large registry analyses, further highlight the need for carefully designed prospective studies, ideally randomized where feasible, to validate the observed findings [14,15].

Taken together, the evidence underscores treosulfan’s growing role in conditioning regimens for MF. It is particularly well-suited for reduced-intensity settings where traditional regimens may be excessively toxic. However, refinement is needed in several areas: dose standardization, identification of optimal partner agents, and better delineation of treosulfan’s efficacy across molecular subtypes of MF. Future research should also investigate post-transplant maintenance strategies to further reduce relapse without increasing toxicity.

## 5. Conclusions

Treosulfan-based conditioning represents a promising and versatile alternative to conventional busulfan-based regimens in the management of myelofibrosis patients undergoing allogeneic hematopoietic stem cell transplantation. The available evidence demonstrates that treosulfan offers a favorable balance of efficacy and tolerability, with high engraftment rates, low non-relapse mortality, and durable disease control. These benefits extend across diverse clinical scenarios, including elderly patients, pediatric populations, second transplants, and alternative donor settings. The reduced organ toxicity and predictable pharmacokinetics of treosulfan make it particularly attractive for patients with comorbidities or advanced age, populations in whom traditional myeloablative conditioning may be prohibitively toxic.

## Figures and Tables

**Table 1:** Summary of Treosulfan Studies in Myelofibrosis.

Study	Population	Regimen	Key Findings	Relapse Risk	Non-Relapse Mortality (NRM)
Large Registry and Multicenter Studie					
Robin et al., 2024 [4]	530 MF patients (457 BU, 73 TREO)	TREO vs. BU-RIC (≤6.4 mg/kg) vs. BU-HD (>6.4 mg/kg)	TREO superior to BU-HD in OS (HR 0.61) and PFS (HR 0.57); NRM lower in TREO; similar relapse rates	No significant difference across groups	Lower in TREO (HR 0.44 vs. BU-HD)
Gagelmann et al., 2024 [3]	1115 MF patients (902 BU, 213 TREO)	TREO/FLU vs. BU/FLU (RIC and high-dose)	4-year OS: 69% with RIC-TREO; high-dose TREO was associated with increased NRM; RIC-TREO showed favorable outcomes	Similar across groups	Increased with high-dose TREO; acceptable with RIC-TREO
Polverelli, et al., 2025 [6]	37 MF patients (Italian multicenter; high-risk cohort)	TREO + thiotepa or melphalan (double-alkylator)	Engraftment: 94.6%; 1-yr OS: 62.2%; PFS: 59.6%; aGVHD II–IV: 21.6%; cGVHD: 18.8%	14.5% at 1 year	33.5% at 1 year; higher with TREO ≥ 47 g/m^2^
Specialized Patient Populations (Elderly, Pediatric, Salvage)					
Gagelmann et al., 2024 [7]	115 MF patients ≥70 years old	19 received TREO + FLU	OS, NRM, and relapse comparable to BU regimens; 96% engraftment; Treo well-tolerated in elderly	7% relapse at 1 year	No significant difference vs. BU
Atagunduz et al., 2020 [8]	33 relapsed MF patients (2nd allo-HCT)	TREO (36–42 g/m^2^) + FLU ± ATG	3-yr OS: 59%, 5-yr DFS: 45%, 5-yr relapse: 16%, day-100 NRM: 16%	16% at 5 years	16% at day 100, 31% at 3 years
Wachowiak et al., 2024 [9]	35 pediatric MF patients	6 received TREO (various combinations)	TREO used mainly in non-MSD setting; tolerated well; no outcome data reported specifically for Treo group	Not reported	Not reported
Single-Center and Pilot Studies					
Claudiani et al., 2014 [10]	14 MF patients (10 MRD, 4 MUD)	TREO + FLU (± low-dose TBI/ATG)	3-yr OS: 54%, 3-yr PFS: 46%, Engraftment: 100%, aGVHD II–IV: 50%, cGVHD: 48%	Not specified	2-yr NRM: 39%, higher in MUD
Campodonico et al., 2023 [11]	14 MF patients with splenomegaly	TREO + FLU ± MEL + Pre-HCT Splenic Irradiation (10 Gy)	3-year OS: approximately 64%; spleen size decreased by 25%; ANC and platelet engraftment occurred in 13/14 patients; PGF occurred in 5 patients, of whom 4 recovered	Not specified	3 deaths (NRM), good tolerability, mostly mild/moderate cGVHD

allo-HCT, allogeneic hematopoietic cell transplantation; ANC, absolute neutrophil count; ATG, antithymocyte globulin; aGVHD, acute graft-versus-host disease; BU, busulfan; cGVHD, chronic graft-versus-host disease; DFS, disease-free survival; FLU, fludarabine; Gy, gray; HCT-CI, hematopoietic cell transplantation-comorbidity index; MEL, melphalan; MRD, matched related donor; MUD, matched unrelated donor; MSD, matched sibling donor; NRM, non-relapse mortality; OS, overall survival; PFS, progression-free survival; PGF, primary graft failure; RIC, reduced-intensity conditioning; TBI, total body irradiation; TREO, treosulfan.

## Data Availability

No new data were created or analyzed in this study.

## Supplementary Materials

The following supporting information can be downloaded at: https://www.mdpi.com/article/10.3390/jcm15114005/s1, Table S1: PRISMA 2020 checklist.
