# Supplementary material for: Treosulfan-Based Conditioning in Allogeneic Stem Cell Transplantation for Myelofibrosis: A Systematic Review
Source: J Clin Med. 2026 May 22;15(11):4005. doi: 10.3390/jcm15114005 (PMC13258572; doi:10.3390/jcm15114005)
Supplement: Supplementary file 1 [file jcm-15-04005-s001.zip › jcm-4280443-supplementary.pdf]

PRISMA 2020 Main Checklist

Topic	No.	Item	Location where item is reported
**TITLE**			
**Title**	1	Identify the report as a systematic review.	Title page: article type and title.
**ABSTRACT**			
**Abstract**	2	See the PRISMA 2020 for Abstracts checklist	
**INTRODUCTION**			
**Rationale**	3	Describe the rationale for the review in the context of existing knowledge.	Introduction, final paragraph.
**Objectives**	4	Provide an explicit statement of the objective(s) or question(s) the review addresses.	Introduction, final paragraph.
**METHODS**			
**Eligibility criteria**	5	Specify the inclusion and exclusion criteria for the review and how studies were grouped for the syntheses.	Methods: eligibility criteria paragraph; Results grouped by study scope and population.
**Information sources**	6	Specify all databases, registers, websites, organisations, reference lists and other sources searched or consulted to identify studies. Specify the date when each source was last searched or consulted.	Methods: literature search paragraph
**Search strategy**	7	Present the full search strategies for all databases, registers and websites, including any filters and limits used.	Methods: keywords and Boolean operators.
**Selection process**	8	Specify the methods used to decide whether a study met the inclusion criteria of the review, including how many reviewers screened each record and each report retrieved, whether they worked independently, and if applicable, details of automation tools used in the process.	Methods: study selection summarized; Figure 1. Reviewer number not specified.

Topic	No.	Item	Location where item is reported
**Data collection process**	9	Specify the methods used to collect data from reports, including how many reviewers collected data from each report, whether they worked independently, any processes for obtaining or confirming data from study investigators, and if applicable, details of automation tools used in the process.	Methods: data extraction paragraph
**Data items**	10a	List and define all outcomes for which data were sought. Specify whether all results that were compatible with each outcome domain in each study were sought (e.g. for all measures, time points, analyses), and if not, the methods used to decide which results to collect.	Methods: OS, PFS, relapse, NRM, engraftment, and GVHD outcomes.
	10b	List and define all other variables for which data were sought (e.g. participant and intervention characteristics, funding sources). Describe any assumptions made about any missing or unclear information.	Methods: patient characteristics, conditioning protocols, donor type, and study design.
**Study risk of bias assessment**	11	Specify the methods used to assess risk of bias in the included studies, including details of the tool(s) used, how many reviewers assessed each study and whether they worked independently, and if applicable, details of automation tools used in the process.	Methods: narrative appraisal of methodological limitations and risk of bias; Discussion: limitations paragraph.
**Effect measures**	12	Specify for each outcome the effect measure(s) (e.g. risk ratio, mean difference) used in the synthesis or presentation of results.	Results/Table 1: HRs, OS/PFS percentages, relapse incidence, NRM, and GVHD rates as reported by included studies.
**Synthesis methods**	13a	Describe the processes used to decide which studies were eligible for each synthesis (e.g. tabulating the study intervention characteristics and comparing against the planned groups for each synthesis (item 5)).	Methods and Results: studies grouped thematically by scope and population.
	13b	Describe any methods required to prepare the data for presentation or synthesis, such as handling of missing summary statistics, or data conversions.	Methods: extracted variables; Results/Table 1.
	13c	Describe any methods used to tabulate or visually display results of individual studies and syntheses.	FIGURE 1/Results/Table 1.

Topic	No.	Item	Location where item is reported
	13d	Describe any methods used to synthesize results and provide a rationale for the choice(s). If meta-analysis was performed, describe the model(s), method(s) to identify the presence and extent of statistical heterogeneity, and software package(s) used.	Methods: descriptive narrative synthesis due to heterogeneity.
	13e	Describe any methods used to explore possible causes of heterogeneity among study results (e.g. subgroup analysis, meta-regression).	Results and Discussion: analyses by registry studies, salvage setting, elderly, pediatric, and pilot studies.
	13f	Describe any sensitivity analyses conducted to assess robustness of the synthesized results.	Not applicable. No pooled meta-analysis was performed.
**Reporting bias assessment**	14	Describe any methods used to assess risk of bias due to missing results in a synthesis (arising from reporting biases).	Not applicable. No quantitative synthesis or funnel-plot assessment was performed.
**Certainty assessment**	15	Describe any methods used to assess certainty (or confidence) in the body of evidence for an outcome.	Methods: formal quantitative certainty grading was not performed; Discussion: limitations.
**RESULTS**			
**Study selection**	16a	Describe the results of the search and selection process, from the number of records identified in the search to the number of studies included in the review, ideally using a flow diagram.	Figure 1: PRISMA 2020 flow diagram.
	16b	Cite studies that might appear to meet the inclusion criteria, but which were excluded, and explain why they were excluded.	Figure 1 gives exclusion categories. Individual excluded studies are not listed.
**Study characteristics**	17	Cite each included study and present its characteristics.	Results/Table 1 and study-specific subsections.
**Risk of bias in studies**	18	Present assessments of risk of bias for each included study.	Discussion: limitations and study-specific caveats; no formal per-study scoring table.

Topic	No.	Item	Location where item is reported
**Results of individual studies**	19	For all outcomes, present, for each study: (a) summary statistics for each group (where appropriate) and (b) an effect estimate and its precision (e.g. confidence/credible interval), ideally using structured tables or plots.	Results/Table 1 and study-specific subsections.
**Results of syntheses**	20a	For each synthesis, briefly summarise the characteristics and risk of bias among contributing studies.	Results and Discussion: thematic synthesis.
	20b	Present results of all statistical syntheses conducted. If meta-analysis was done, present for each the summary estimate and its precision (e.g. confidence/credible interval) and measures of statistical heterogeneity. If comparing groups, describe the direction of the effect.	Not applicable. No pooled statistical synthesis was conducted.
	20c	Present results of all investigations of possible causes of heterogeneity among study results.	Discussion: dose intensity, donor type, age, pediatric setting, salvage transplantation, and double-alkylator regimens.
	20d	Present results of all sensitivity analyses conducted to assess the robustness of the synthesized results.	Not applicable. No pooled synthesis was conducted.
**Reporting biases**	21	Present assessments of risk of bias due to missing results (arising from reporting biases) for each synthesis assessed.	Not applicable. No quantitative reporting-bias assessment was conducted.
**Certainty of evidence**	22	Present assessments of certainty (or confidence) in the body of evidence for each outcome assessed.	Discussion: limitations; formal certainty grading not performed.
**DISCUSSION**			
**Discussion**	23a	Provide a general interpretation of the results in the context of other evidence.	Discussion, opening and subsequent paragraphs.
	23b	Discuss any limitations of the evidence included in the review.	Discussion: limitations paragraph.
	23c	Discuss any limitations of the review processes used.	Methods and Discussion: narrative synthesis, no meta-analysis, potential overlap.

Topic	No.	Item	Location where item is reported
	23d	Discuss implications of the results for practice, policy, and future research.	Discussion and Conclusion.
**OTHER INFORMATION**			
**Registration and protocol**	24a	Provide registration information for the review, including register name and registration number, or state that the review was not registered.	Methods: protocol was not prospectively registered.
	24b	Indicate where the review protocol can be accessed, or state that a protocol was not prepared.	Methods: protocol was not prospectively registered.
	24c	Describe and explain any amendments to information provided at registration or in the protocol.	Not applicable. No registered protocol.
**Support**	25	Describe sources of financial or non-financial support for the review, and the role of the funders or sponsors in the review.	Back matter: Funding.
**Competing interests**	26	Declare any competing interests of review authors.	Back matter: Conflicts of Interest.
**Availability of data, code and other materials**	27	Report which of the following are publicly available and where they can be found: template data collection forms; data extracted from included studies; data used for all analyses; analytic code; any other materials used in the review.	Back matter: Data Availability Statement; no new data were created or analyzed.

PRIMSA Abstract Checklist

Topic	No.	Item	Reported?
**TITLE**			
**Title**	1	Identify the report as a systematic review.	Yes
**BACKGROUND**			
**Objectives**	2	Provide an explicit statement of the main objective(s) or question(s) the review addresses.	Yes
**METHODS**			
**Eligibility criteria**	3	Specify the inclusion and exclusion criteria for the review.	Yes
**Information sources**	4	Specify the information sources (e.g. databases, registers) used to identify studies and the date when each was last searched.	Yes
**Risk of bias**	5	Specify the methods used to assess risk of bias in the included studies.	No
**Synthesis of results**	6	Specify the methods used to present and synthesize results.	Yes
**RESULTS**			
**Included studies**	7	Give the total number of included studies and participants and summarise relevant characteristics of studies.	Yes
**Synthesis of results**	8	Present results for main outcomes, preferably indicating the number of included studies and participants for each. If meta-analysis was done, report the summary estimate and confidence/credible interval. If comparing groups, indicate the direction of the effect (i.e. which group is favoured).	Yes
**DISCUSSION**			
**Limitations of evidence**	9	Provide a brief summary of the limitations of the evidence included in the review (e.g. study risk of bias, inconsistency and imprecision).	Yes

Topic	No.	Item	Reported?
**Interpretation**	10	Provide a general interpretation of the results and important implications.	Yes
**OTHER**			
**Funding**	11	Specify the primary source of funding for the review.	Yes
**Registration**	12	Provide the register name and registration number.	No

From: Page MJ, McKenzie JE, Bossuyt PM, Boutron I, Hoffmann TC, Mulrow CD, et al. The PRISMA 2020 statement: an updated guideline for reporting systematic reviews. MetaArXiv. 2020, September 14. DOI: 10.31222/osf.io/v7gm2. For more information, visit: www.prisma-statement.org
